# Enzyme Cascade Electrode Reactions with Nanomaterials and Their Applicability towards Biosensor and Biofuel Cells

**DOI:** 10.3390/bios13121018

**Published:** 2023-12-07

**Authors:** Shalini devi Kalyana Sundaram, Md. Motaher Hossain, Muhammad Rezki, Kotoko Ariga, Seiya Tsujimura

**Affiliations:** Division of Material Science, Faculty of Pure and Applied Science, University of Tsukuba, 1-1-1, Tennodai, Tsukuba 305-5358, Japan; k.shalinidevi.fp@u.tsukuba.ac.jp (S.d.K.S.); s2030072@u.tsukuba.ac.jp (M.M.H.); s2236006@u.tsukuba.ac.jp (M.R.); s2320322@u.tsukuba.ac.jp (K.A.)

**Keywords:** nanomaterials, enzyme cascade, biosensor, biofuel cell, electrode

## Abstract

Nanomaterials, including carbon nanotubes, graphene oxide, metal–organic frameworks, metal nanoparticles, and porous carbon, play a crucial role as efficient carriers to enhance enzyme activity through substrate channeling while improving enzyme stability and reusability. However, there are significant debates surrounding aspects such as enzyme orientation, enzyme loading, retention of enzyme activity, and immobilization techniques. Consequently, these subjects have become the focus of intensive research in the realm of multi-enzyme cascade reactions. Researchers have undertaken the challenge of creating functional in vitro multi-enzyme systems, drawing inspiration from natural multi-enzyme processes within living organisms. Substantial progress has been achieved in designing multi-step reactions that harness the synthetic capabilities of various enzymes, particularly in applications such as biomarker detection (e.g., biosensors) and the development of biofuel cells. This review provides an overview of recent developments in concurrent and sequential approaches involving two or more enzymes in sequence. It delves into the intricacies of multi-enzyme cascade reactions conducted on nanostructured electrodes, addressing both the challenges encountered and the innovative solutions devised in this field.

## 1. Introduction

Natural catalysts include complete cells or enzymes, commonly referred to as “biocatalysts”. In particular, the emergence of multi-enzyme biocatalysts, capable of orchestrating numerous biocatalytic reactions, has bridged the gap between single-enzyme catalysis and whole-cell catalysis. This development has had a profound impact on various fields such as biofuel cells (BFCs), biosensors, and biomedical engineering [1]. These multi-enzyme cascades can be categorized based on the final product and intermediates formed during the reactions: linear cascades [2,3], parallel cascades [4,5], orthogonal cascades [6,7], and cyclic cascades [8]. Each cascade begins with the generation of a reactive intermediate, and this categorization has expedited the creation of novel synthetic multi-enzymatic pathways [9]. Designing enzyme cascade reactions can be approached in three major ways: fusion of enzymes [10], co-immobilization of enzymes [11], and enzyme–scaffold complexes [12]. When combined with various nanomaterials like carbon nanotubes (CNTs), graphene oxide (GO), metal–organic frameworks (MOFs), MXenes, metal nanoparticles, or bio and organic molecules such as DNA, crosslinkers, and redox polymers, these techniques enhance surface area, spatial control, substrate mass transfer, stability, catalytic activity, and the retention of co-immobilized enzymes [13]. Functionalizing nanomaterials with specific ligands or molecules can also introduce selectivity to enzyme cascades, improving their specificity.

Efforts have been made to replicate these enzymatic cascade events in vitro for use in chemical synthesis. In biosensors, the recognition element that interacts with the target analyte comprises enzymes, antibodies, aptamers, and similar components [14]. Typically, the first enzyme involved in the reaction with the analyte or yielding a product related to the analyte is closely linked to this recognition element. In some cascade biosensors, multiple enzymes participate in successive reactions, necessitating intermediary enzymes. These intermediaries aid in converting the output of the first enzyme into a more detectable form or one that can be utilized by a second enzyme. Finally, the last enzyme in the cascade catalyzes a reaction that produces a detectable signal, often through changes in color, electrical conductivity, or the release of a fluorescent or electrochemical signal [15]. Hence, this study provides an overview of the role of nanomaterials in enzyme cascade reactions within biosensors and offers valuable insights into their practical applications.

BFCs based on enzyme cascade reactions represent a promising avenue for clean and sustainable energy conversion. They hold the potential to revolutionize energy generation by harnessing remarkable power efficiency while minimizing environmental impact [16]. High-energy density compounds can be electrochemically oxidized by biological catalysts to produce electricity. However, there are constraints on the energy density of the resulting BFCs. This limitation arises because single enzymes, typically used in bioanode designs, can only partially oxidize biofuels via a two-electron process, leaving unused electrons [17]. Before the incorporation of nanomaterials, BFCs relied solely on a variety of enzymes and their respective reaction activities. For example, Palmore et al. offered valuable insights into the complete oxidation of methanol to CO_2_ using NAD-dependent dehydrogenases and proposed strategies to reduce voltage losses in BFCs caused by activation overpotentials. They demonstrated the effectiveness of benzyl viologen-mediated oxidation of NADH to NAD^+^ by diaphorase in constructing BFCs [18].

Moving beyond single enzymes, the utilization of two or more enzymes offers advantages such as greater energy densities. Additionally, quick electron transfer reactions occur among multiple enzymes, especially when combined with nanomaterials possessing various properties. This synergy results in enhanced current and power densities. This review explores the latest developments and promising future directions in the fields of biosensors and BFCs, with a particular focus on various nanomaterials, DNA, crosslinker support, redox polymers, and polymers/hydrogels for multi-enzyme immobilization, providing an in-depth discussion of these topics.

## 2. Nanomaterials—An Emerging Tool for Enzyme Cascade Reactions

### 2.1. Porous Carbon

**Figure 1:**
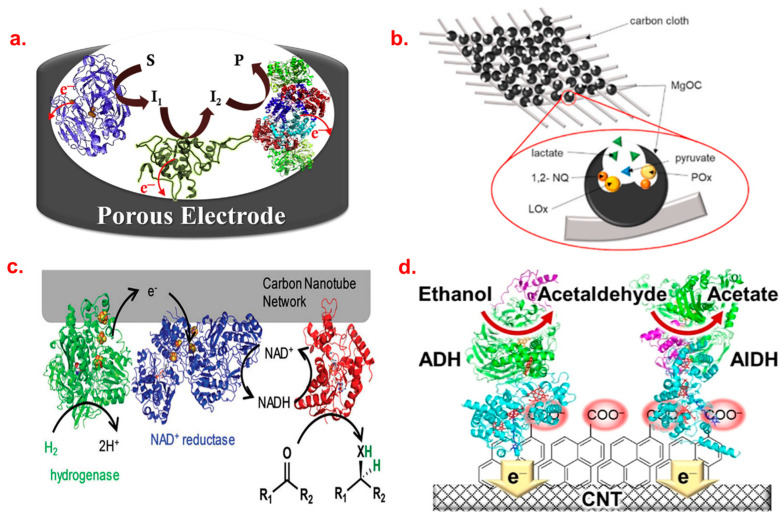
Schematic illustration of enzyme incorporation into the porous carbon material, where S, I, and P represent substrate, intermediate, and product, respectively (**a**) (reprinted with permission from Ref. [20], Copyright 2021, Elsevier) and its application towards BFC, where NQ represents naphthoquinone. (**b**) (reprinted with permission from Ref. [25], Copyright 2023, Elsevier). Similarly, the hydrogenase and reductase-loaded MWCNT for H_2_-driven NADH production is illustrated [27] (**c**), and the role of MWNCT in constructing a biofuel is depicted (**d**) (reprinted with permission from Ref. [28], Copyright 2023, ACS).

Porous carbon materials have been attracting attention for their applicability for scaffolds of enzyme immobilization [19]. They have high surface area, good electrical conductivity, and outstanding biocompatibility, all of which are important properties in fabricating bioelectrochemical devices such as BFCs and biosensors. The development of biodevices based on direct electron transfer (DET) requires an adequate number of immobilized enzymes, ideally with an acceptable orientation to facilitate the electron exchange with the electrode and easy accessibility of the substrate to the active site. Adequate enzyme immobilization has emerged as a crucial step in the process [20], as shown in Figure 1a, which is attained via the mesoporous carbon materials, MgO-templated carbon (MgOC), since they are known to have a high specific surface area and an excellent capacity for enzyme adsorption. The size and volume of mesopores and micropores can be tuned by selecting the MgO precursor and carbon precursor, respectively [21]. Having tunable pore size is a great advantage, considering the application for biodevices, because the pore size of MgOC can affect enzymatic and biocatalytic properties in the system. According to Mazurenko et al., the catalytic current increased when the pore size was larger than the enzyme size, attributed to high enzyme loading, while the enzyme stability at a higher temperature was enhanced by the smaller pore size closer to the enzyme size. The improvement in enzyme stability is probably because of the stronger interactions between MgOC and enzymes [22]. Because of these attractive characteristics, MgOC has been utilized in BFCs. Niiyama et al. developed a glucose/O_2_ BFC using MgOC-carbon textile composite electrodes and applying flavin adenine dinucleotide-dependent glucose dehydrogenase (FAD-GDH) as the anode and bilirubin oxidase (BOD) as the cathode. The maximum output power density of this BFC was 2 mW cm^−2^ at 0.4 V [23].

Considering the advantages of the use of mesoporous carbon materials, especially MgOC, in bioelectrocatalysis, it is expected that the performance of BFCs can improve by combining enzyme cascade reactions with mesoporous carbon electrodes. Shitanda et al. applied a three-enzyme cascade reaction involving lactate oxidase (LOx), pyruvate decarboxylase (PDC), and pyrroloquinoline quinone-dependent aldehyde dehydrogenase (ALDH) to oxidation at the bioanode [24]. Four electrons were produced through one cycle of this cascade reaction. All the enzymes were immobilized on the surface of a carbon cloth (CC) with an MgOC electrode via physical adsorption. A BOD-modified electrode was used as the biocathode. The BFC with an LOx-modified bioanode, with two electrons per reaction cycle, produced a current density of about 0.3 mA cm^−2^ at 600 s, while the BFC using LOx/PDC/ALDH with four electrons per reaction exhibited approximately 1 mA cm^−2^ of current density in the same conditions. In the LOx/PDC/ALDH cascade system, the existence of pyruvate prevents a lack of fuel near the electrode surface even after most lactate is consumed. This condition can solve the problem of the diffusion speed of lactate, which is the rate-limiting step in BFCs using only LOx. Shitanda’s group also fabricated a high-performance BFC which employs a BOD-dropped biocathode and a two-enzyme bioanode using LOx and pyruvate oxidase (POx) [25], as shown in Figure 1b. Both the electrodes are made of MgOC-modified carbon cloth, and the LOx and POx are immobilized inside the pores. This BFC achieved the maximum power and current density of 1.75 mW cm^−2^ and 7.25 mA cm^−2^, respectively. The combination of mesoporous carbon materials and enzyme cascade reactions is also used for biosensors. For example, Kawai et al. used Ketjen Black (KB), a kind of conductive carbon black, to modify a gold (Au) microdisk electrode for the immobilization of POx and horseradish peroxidase (HRP) as a pyruvate sensor [26]. By using this strategy, a linear current response of up to 600 µM of pyruvate was achieved. This sensor does not require mediators because it uses DET-type bioelectrocatalysis.

### 2.2. Carbon Nanomaterials

CNTs are nanosized circular tubes with suitable characteristics for enzyme immobilization such as adjustable surface entities, astonishing surface-volume area, good electrical conductivity, and mechanical, chemical, and thermal stability [11,29]. A series of research on enzyme cascade reactions was conducted with these nanotubes as electrode platforms for constructing biosensors and BFCs, which is as follows. Zor et al. demonstrated the potential for biocatalytic hydrogenations using enzymes immobilized on a CNT-lined quartz column (CNC) in continuous flow of both hydrogenase and reductase [27], as seen in Figure 1c. The sequential co-immobilization of α-amylase (AA) and glucoamylase (GluA) on a silica microsphere immobilized on CNTs provided excellent stability and strong adsorption of enzymes, and improved catalytic activity was discussed by Du et al. [30]. In terms of application, Lang et al. developed an enzyme cascade system exhibiting an H_2_O_2_ biosensor and starch/O_2_ biofuel activity. It was constructed via the co-immobilization of two enzymes, namely, GA and GOD, on a GCE modified with multi-walled carbon nanotubes (MWCNTs) using a chemical crosslinking approach. The crosslinker, glutaraldehyde (GA), and the blocking agent, bovine serum albumin (BSA), were used to prevent the leaching of enzymes. FAD present in the GOD promotes the DET between the GOD- and CNT-modified electrodes. Using the GA/GOD/MWCNTs/GCE electrode as the bioanode and laccase/MWCNTs/GCE as the biocathode, a starch/O_2_ BFC was constructed. This cell displayed open circuit voltage up to approximately 0.53 V and a maximum power density of 8.15 μW cm^−2^ at 0.31 V, which is comparable to the performance of existing glucose/O_2_-based BFCs [31]. Similarly, Zhang et al. utilized a screen-printed electrode (SPE) modified with two enzymes, invertase (INV) and GDH, to develop a MWCNT/INV/GDH electrode, along with the use of redox mediators such as methylene green (MG) in combination with [Ni(phendion)(phen)]Cl_2_ complex for the detection of nicotinamide adenine dinucleotide (NADH) at a lower overpotential. This modified electrode was alternately built upon DNA nanocomposites and polyethyleneimine (PEI) to create a bioanode. While MWCNTs promoted effective electron transfer from sucrose oxidation and increased current density, the layer-by-layer architecture showed advantages for sequential enzymatic reactions that encouraged the efficient penetration of substrate and products in a cascade system. The BFC produced a greater power density (W cm^−2^) with a GCE bioanode modified with an MG or Ni complex, with 145.8% and 130.11% enhancements in comparison to an SPE bioanode [32]. In another work, Contaldo et al. immobilized copper-containing nitrite reductase (NiR) on MWCNT electrodes modified with 4-(1-pyren) butyric acid adamantylamide [33]. This work explains that the role of 4-(1-pyren) butyric acid adamantylamine is to enhance the hydrophobicity of CNT sidewalls; therefore, the hydrophobic part of the modified CNTs can strongly interact with the hydrophobic substrate pocket of NiR. Hence, the main driving force of this immobilization is hydrophobic interactions between NiR and the surface of MWCNTs. The electrode showed a high bioelectrochemical reduction of nitrite with a current density of 1.41 mA cm^−2^. Franco et al. prepared an ADH/ALDH-immobilized bienzymatic bioanode using carbon paper containing electropolymerized MG/MWCNTs [34]. They carried out power density measurements and found that the ADH/ALDH bioanode produced a power density which was 6.3 times higher than that obtained from the ADH monoenzymatic system. Later, Adachi et al. demonstrated that by immobilizing the enzymes on MWCNTs functionalized with 1-pyrene carboxylic acid, ADH/ALDH bienzymatic bioanode can be produced. Recently, high electricity was generated by an ethanol/air BFC using this bioanode, which showed a maximum current density of 2.69 ± 0.09 mA cm^−2^ and a maximum power output of 0.48 ± 0.01 mW cm^−2^ at 0.366 ± 0.004 V [28], as shown in Figure 1d. Chansaenpak et al. fabricated a BFC using a graphite electrode, which was modified with MWCNTs, as the cathode [35]. The modified CNT electrode was immobilized with 1-pyrenebutyric acid N-hydroxysuccinimide ester, HRP, and glucose oxidase (GOx), sequentially. The bioanode was a reduced graphene oxide (rGO)-modified glassy carbon electrode which was immobilized with poly(toluidine blue O)-modified NAD-dependent GDH. The BFC showed 31.3 µW cm^−2^ of a maximum power density at a potential of 0.3 V with 40 mM glucose. The proposed BFC was investigated for the detection of glucose as a self-powered biosensor. By using a nonlinear calibration, the sensor could determine glucose concentration in the range of 0.1–7.0 mM, which is a suitable level of detection for common biofluids such as blood. It is considered that such high sensitivity as a biosensor was achieved via the increased potential of the cell (OCV = 0.65 V), resulting from the enzymatic cascade reaction on the cathode. Thus, CNTs are proven to be widely modifiable, and this has a greater advantage as an enzyme immobilization platform for designing various biosensors and BFCs. Some examples of enzyme cascade systems on CNTs and MgOC are summarized in Table 1.

### 2.3. Graphene and Graphene Oxide (GO/rGO)

**Figure 2:**
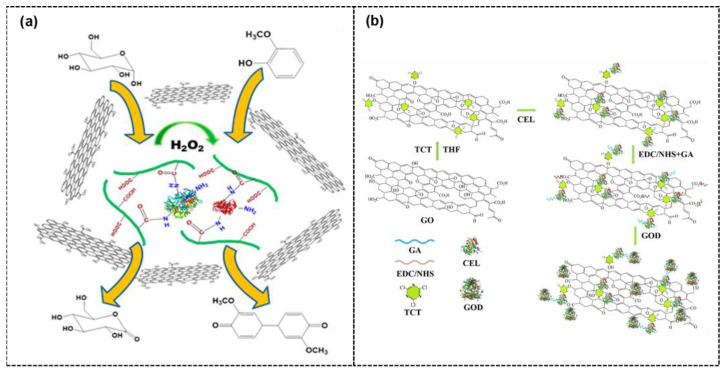
Enzyme cascade system via GO with model enzymes GOx and HRP with poly(acrylic acid) as the model polymer within a network to function (**a**) (reprinted with permission from Ref. [40], Copyright 2015, American Chemical Society) and a scheme for co-immobilization of CEL and GOD (**b**) (reprinted with permission from Ref. [42], Copyright 2019, Wiley).

Since the GO surface’s functional groups are easily accessible for reactions involving biomolecules, protein immobilization over graphene sheets is easily accessible, and hence, it plays a vital role in biosensor applications [39]. Zore et al. investigated the creation of a stable, high-temperature, and pH-resistant catalytic system for glucose oxidation, achieved by combining two enzymes, GOx and HRP, within a polymer network via simple physical adsorption of graphene oxide (GO) nanoplates [40] (Figure 2a). The destabilization of GOx higher than 50 °C was retained via polymer adsorption with GO. The role of GO–polyethylene glycol (PEG) served as a stable platform for delivering biologically active compounds, such as enzymes, to cells in this work. This can enable precise control over biological processes. Adsorption onto a 2D nanolayered material after enzyme–polymer conjugation results in increased substrate channeling and high enzyme cascade stability [40]. Similarly, GOx and GA were immobilized on chemically reduced graphene oxide (CRGO) to create a multi-enzyme microsystem reported by Zhao et al. The stability of the multi-enzyme biocatalyst immobilized on CRGO was significantly improved compared to a system using GO as the carrier. The starch-to-gluconic acid process was carried out in one pot using this multi-enzyme microsystem as a biocatalyst, and the gluconic acid yield could reach 82% in under two hours. These findings showed that the unique method of building biomicrosystems with several enzymes on 2D CRGO via noncovalent bonds to carry out some complex conversions was feasible [41]. Zhang et al. investigated a step-by-step method to fabricate the biocatalytic system that involves GOx and cellulase (CEL) for the effective conversion of glucose to gluconic acid and carboxymethyl cellulose (CMC) [42]. Cyanuric chloride (TCT), EDC-NHS, GA, and tetrahydrofuran were used for GO activation and for cleaning purposes during the fabrication of the electrodes. The results demonstrated the viability of the technique of constructing a biomicrosystem with CEL and GOD immobilized on GO via covalent bonds in order to convert CMC to gluconic acid in a single step (Figure 2b). The enzyme cascade applications over GO/rGO need deeper study and mechanistic features which would be of growing interest in future.

## 3. DNA Scaffold for Multi-Enzyme Cascade System

**Figure 3:**
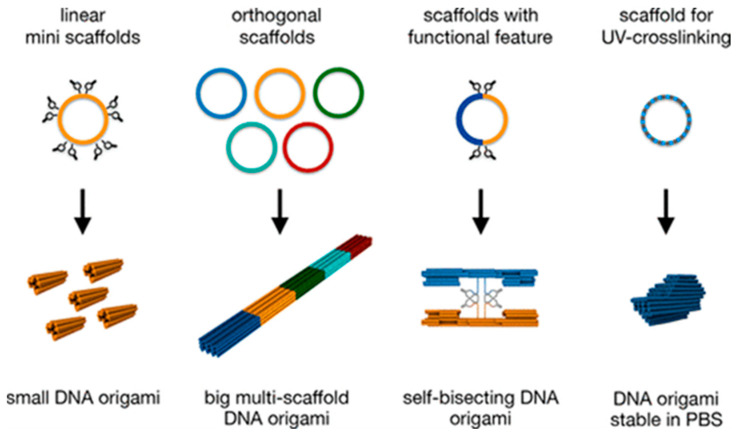
The scaffold vector for the design of defined DNA origami (reprinted with permission from Ref. [49], Copyright 2019, American Chemical Society).

**Figure 4:**
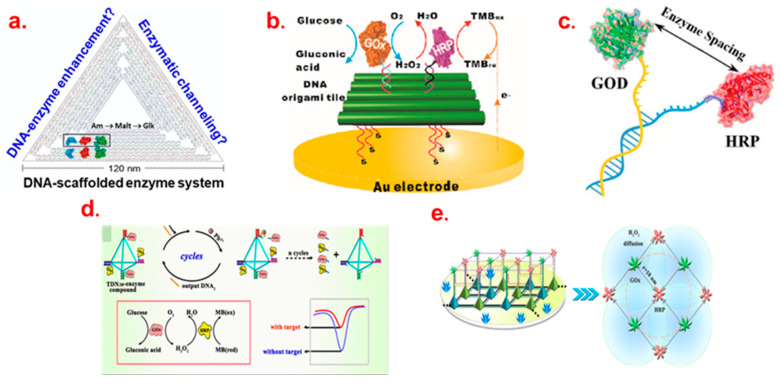
(**a**) Triangle-shaped (reprinted with permission from Ref. [67], Copyright 2019, American Chemical Society), (**b**) DNA tiles [68], (**c**) Y-shaped [69], (**d**) tetrahedron (reprinted with permission from Ref. [70], Copyright 2019, American Chemical Society), and (**e**) tetrahedron-lattice (reprinted with permission from Ref. [71], Copyright 2020, American Chemical Society).

A multi-enzyme cascade reaction is involved in the production of energy and the complete metabolism of a biomolecule, in which the reaction efficiency of the enzyme depends on the regulation of distance among the enzymes on the immobilized scaffold [43]. Conventionally, the enzyme cascade system co-immobilizes via adsorption, covalent binding, polymer hydrogel, nanomaterial, and crosslinking; all of the methods are based on random co-immobilization, in which it is difficult to accurately control the spatial position of the enzymes and high-efficiency system [44]. In order to solve this problem, various attempts are developed such as encapsulation [45], layer-by-layer co-immobilization, and nanochambers [46]. Among the various strategies for the control of spatial position enzymes, DNA is the attractive biocompatible biomaterial used to construct nanoscale engineered structures allowed in various applications [47]. The scaffold DNA sequences have determined many targeted properties to modify the DNA nanostructure via other molecules for introducing DNA-based functional architectures [48,49,50,51,52]. Usually, the DNA scaffold is used to manipulate a biomolecule such as a protein via engrafting to allow for robust molecule access [53]. Particularly, the assemblies of DNA and protein lead to the generation of structural and functional biomaterial [54,55,56]. Recently, the DNA nanostructured material has been developed impressively with a variety of structures through the rational design of base pairing method [57,58]. By taking these properties, the DNA origami nanostructure has precisely led to several applications, including multi-enzyme [59], nanoactuator [60], protein–DNA interactions [61,62], and aptamer [63]. However, the reaction efficiency of the enzyme depends on the regulation of the distance of the multi-enzyme on the immobilized DNA scaffold [64,65,66]. It is difficult to attain complete control of inter-enzyme distance, owing to their reliably designed DNA scaffold. Various examples have been explored with the design of DNA origami, as shown in Figure 3, such as when Wilner et al. topologically organized the enzyme cascade system by the self-assembly of a DNA scaffold, namely, “hexagon-shaped”. This work successfully controls the topology of DNA, which controls the relative position of GOx and HRP [59]. Klein et al. constructed a triangle-shaped DNA scaffold for the immobilization of three-enzyme amylase, maltase (MAL), and glucokinase; the DNA nanostructured scaffold demonstrated the sequential catalysis of the multi-enzyme by exploiting the channeling effects, as in Figure 4a [67]. Zhilei et al. assembled and named a “DNA origami tile” for the two enzymatic GOx and HRP systems for electrochemical devices. This work mainly tuned the distance between the GOx and HRP to control the electrical potential substrate/product flow through the cascade system, as in Figure 4b [68]. Recently, Chu et al. developed a Y-shaped DNA scaffold for the co-immobilization of GOx and HRP. This work successfully reduces inter-enzyme spacing’s distance to improve the cascade activity to 967 U mg^−1^ at 13.6 nm [69], as seen in Figure 4d. Similarly, Wang et al. improved the controllability and biocompatibility of a scleroid skeleton by engineering a DNA tetrahedron scaffold that controls the two-enzyme distance by pairing Gox and HRP. This work precisely controls the distance allowed for DNA detection by 3 fM LOD, as in Figure 4d,e [70,71].

### DNA Scaffold for the Multi-Enzyme BFC and Biosensor

**Figure 5:**
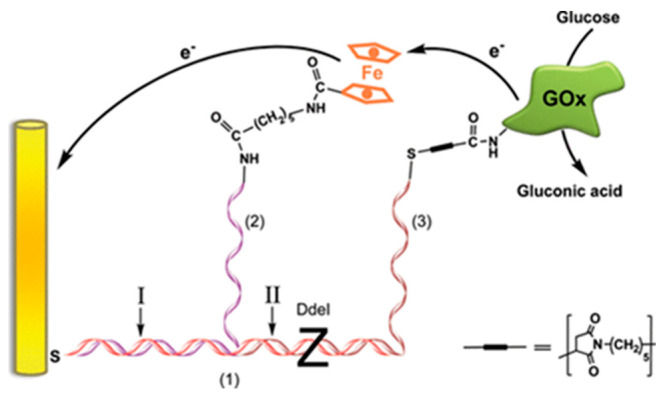
DNA scaffold enhances the electrical contacting of GOx with an electrode via the ordered hybridization of ferrocene (reprinted with permission from [74], Copyright 2009, American Chemical Society).

**Figure 6:**
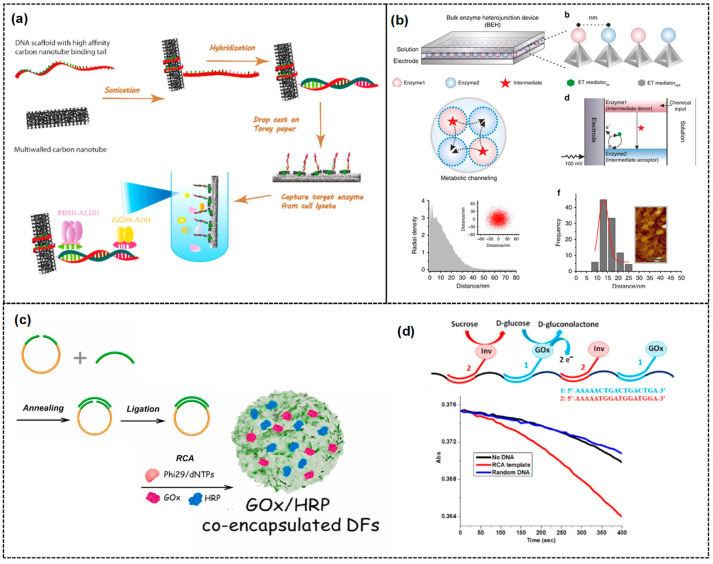
DNA scaffold for multi-enzyme cascade employed for the generation of power based on the anode (**a**) ALD/DLD (reprinted with permission from [75], Copyright 2017, American Chemical Society), (**b**) TDN for biomarker detection (reprinted with permission from [76], Copyright 2020, Nature), (**c**) RCA for glucose detection (reprinted with permission from [77], Copyright 2021, Elsevier), and (**d**) the detection of biorecognition molecule (reprinted with permission from [78], Copyright 2014, IOP Science).

The enzymatic BFC is a promising tool for energy conversion [72]; however, it has the limitation of producing low energy density for the incomplete oxidation of fuel [73]. To address this issue, a multi-enzyme cascade system has been developed for deep or complete oxidation of fuel; it helps to enhance the high energy density of BFC to develop an effective energy device. Recently, the DNA scaffold has become a powerful tool for increasing multi-enzyme catalytic activity for power generation because DNA provides a programmable and versatile platform to precisely immobilize the various enzymes in a nanometer scale. By taking advantage of this, various researchers have used DNA origami to construct enzymatic biofuel cells to improve power generation. In the work of Gilad et al., the first DNA scaffold was utilized as an electrode platform towards the improvement of electrical contact between the GOx and mediator [74], as shown in Figure 5. Later, The Minteer group improved substrate channeling by utilizing DNA templated with two enzymes, namely, alcohol dehydrogenase (ADH) and ALDH, for biofuel cells. The enzyme was tagged with a zinc finger domain as well as anchored with a DNA-modified carbon nanotube (Figure 6a) [75]. The constructed anode-based system was improved to the maximum power generation of 24 μW cm^–2^. Recently, Song et al. constructed a 3D tetrahedral DNA nanostructure (TDN) framework for the sensor platform (Figure 6b), leading to a uniform biorecognition layer for biomarker detection with remarkable sensitivity [76]. This work engineered the bulk enzyme heterojunction strategy with TDN for sarcosine oxidase (SOx) and HRP cascade employed for electrochemical biomarker detection. They successfully created an enzyme cascade system in mm scale with an enzyme pair with a 10 nm interface. Yongcun et al. constructed a high-performance cascade system by developing an RCA-based technique toward a microsized DNA flower (DF) (Figure 6c), which captures two enzymes, GOx and HRP [77]. The interaction between the enzyme cascade and DF is bridged via magnesium from the enzyme and phosphate backbone on DF. The DNA scaffold shows an excellent biointerface with high detection capabilities of LOD 3.3 µM. Ding et al. designed a new DNA scaffold such as tetrahedron (TDN) [70] and TDN-lattice [71] in order to make a GOx/HRP cascade system for the detection of thrombin and DNA, respectively. This work successfully controls the distance between the enzymes to improve the efficiency of the cascade reaction. Khiem et al. utilized a DNA scaffold to immobilize the invertase and GOx cascade system to improve the catalytic efficiency of sucrose (Figure 6d) [78]. This work has successfully assembled two enzymes on rolling circle amplification (RCA) to improve power generation by 75% more than an enzyme in solution. Table 2 reviews the comparison of BFCs and biosensors based on a multi-enzyme cascade system via a DNA scaffold. 

## 4. Involvement of MOF Nanomaterial in Enzymes Cascade Reaction

**Scheme 1:**
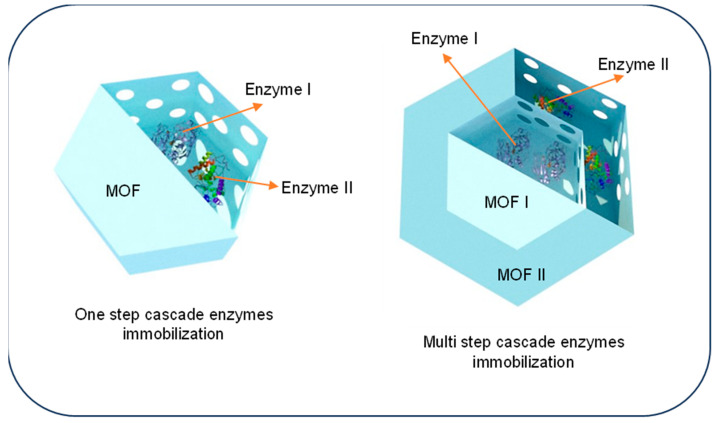
Multi-enzyme immobilization strategy in MOFs to support cascade reaction.

Numerous studies have documented the effectiveness of enzymatic cascade systems supported by MOFs for a variety of applications [80]. As generally known, an MOF is an organic-inorganic hybrid material that is built from the coordination bonding of various metal ions and organic linkers that form a porous crystalline structure [81]. The ability to be synthesized under mild conditions, high chemical and temperature stability, tunable structure, permanent porosity, and large surface area are the main reasons for the MOF family becoming a favorable platform for enzyme modification. In the MOF–cascade enzyme preparation process, the enzymes co-immobilization method mainly can be divided into two different approaches: first, one-step co-immobilization, and second, multi-step co-immobilization. In the first method, two or more enzymes will be mixed at the same time in one pot with the MOF precursors, while in the second method, one enzyme will be immobilized first, followed by the incorporation of the second enzyme, the third enzyme, and so on. This strategy will produce a layer-by-layer enzyme–MOF architecture that will give some advantages in certain applications (Scheme 1).

### 4.1. One-Step Co-Immobilization of Enzymes Using MOF

**Figure 7:**
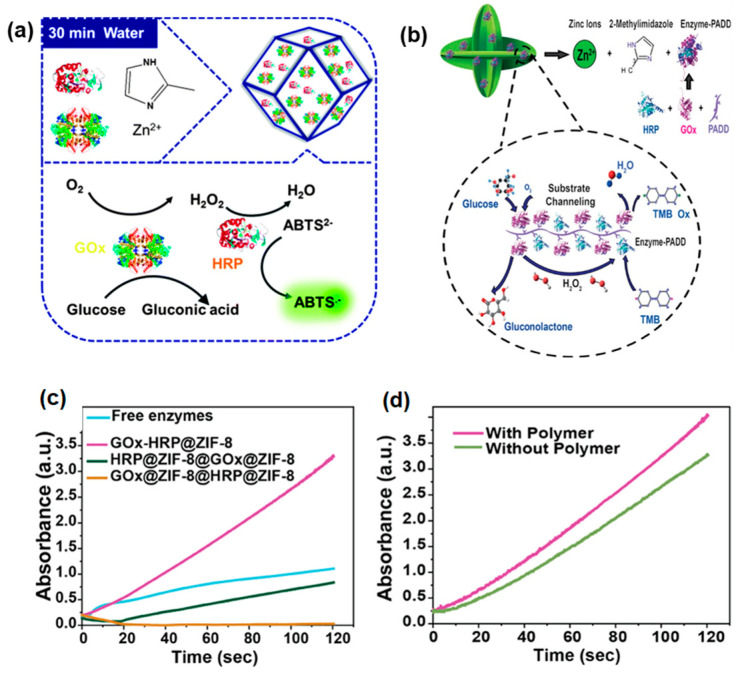
(**a**) One-step co-immobilization enzymes cascade in ZIF-8 (reprinted with permission from [82], Copyright 2015, Royal Chemical Society), (**b**) one-step co-immobilization enzymes cascade involving PADD polymer, (**c**) catalytic activity comparison of free enzyme and different types of immobilized cascade enzymes, (**d**) catalytic activity comparison of immobilized enzymes with and without PADD polymer (reprinted with permission from [84], Copyright 2023, American Chemical Society).

Early research on immobilizing several enzymes in MOFs was mostly centered on a one-step synthesis approach. Liu et al. simultaneously immobilized GOx and HRP in a one-pot synthesis process along with Zn^2+^ and 2-Mim. As a common cascade system with GOx and HRP, the glucose detection mechanism is based on a colorimetric method in the help of 2,2′-azino-bis(3-ethylbenzothiazoline-6-sulfonic acid) (ABTS) (Figure 7a). This work pioneered the co-immobilization of enzymes inside of an MOF structure and inspired a lot of researchers [82]. Chen et al. reported a cascade system involving three different enzymes, β-galactosidase (β-Gal), GOx, and HRP, prepared via a one-pot co-immobilization process in ZIF-8. The cascade reaction was initiated by lactose as a substrate. In detail, the immobilized β-Gal catalyzed the hydrolysis of lactose to galactose and glucose, and then the glucose was oxidized by GOx to produce gluconate and H_2_O_2_. The H_2_O_2_ was used by the HRP to catalyze the oxidation of amplex red to resorufin, which can be observed through a colorimetry method. Compared to the mixture of these three enzymes in a free system, the embedded enzymes in the MOF show higher catalytic activity by 5.3 fold. This result is because the confined cascade system has more concentrated product from each enzymatic catalysis process, which is then used as feed for the subsequent reaction [83]. Still in the same work, a cascade system established from NAD-dependent enzymes was also reported. A phenylboronic acid-conjugated poly(allylamine) polymer was used to covalently bound the NAD^+^ molecule. In this study, ADH, NAD^+^-conjugated polymer, and LDH were simultaneously immobilized in the ZIF-8 structure. Ethanol and pyruvate were the substrates in this cascade system. In further detail, the immobilized ADH initiated the reduction of NAD^+^-conjugated polymer to NADH-conjugated polymer by catalyzing the oxidation of ethanol to acetyl aldehyde, while the LDH converted the pyruvate to lactate. The rate of reduction of pyruvate to lactate is highly dependent on the individual components in the cascade system [83]. Recently, one-step immobilization of GOx and HRP in ZIF-8 involving a polymer was also reported by Fernando et al. In this case, poly-(acrylamide-co-diallyl dimethylammonium chloride) (PADD), a cationic polymer with an amine functional group, was used to modulate the enzymes immobilization process (Figure 7b). In the glucose detection process, the cascade reaction involves 3,3′,5,5′-tetramethylbenzidine (TMB) as chromogenic substances. The production of H_2_O_2_ from the glucose–GOx reaction was then used by the HRP to oxidize the TMB, which can be quantitively detected using a spectrophotometry method. In this study, they found that compared to subsequent immobilization, the one-step immobilization technique results in higher cascade enzymatic activity (Figure 7c). The utilization of PADD also increases the enzyme catalytic activity, 1.5 higher compared to the immobilized enzymes without PADD (Figure 7d) [84]. 

The covalent immobilization of enzymes on the surface of functionalized MOFs has also been described, in addition to immobilization through encapsulation or co-precipitation. The advantage of using this strategy is efficient substrate diffusion due to a barrierless immobilization approach. For example, Xia et al. synthesize NH_2_-MIL-101 from Fe^3+^ metal ions and NH_2_-BDC linkers. The available NH_2_ site on the MOF surface was crosslinked to the amine group of the enzyme using glutaraldehyde. Palmitic acid was used to create defects on the NH_2_-MIL-101 surface, which enhances the peroxidase-like activity of this material. Two different enzymes, GOx and β-Gal, were immobilized to carry out the colorimetric detection of lactose. In the presence of lactose, the β-Gal will catalyze this substrate to galactose and glucose, then, the glucose will be oxidized by the GOx and produce H_2_O_2_, and finally, the peroxidase-like activity of the NH_2_-MIL-101 catalyzes the oxidation of TMB in the presence of H_2_O_2_ [85].

### 4.2. Multi-Step Co-Immobilization of Enzymes Using MOF

**Figure 8:**
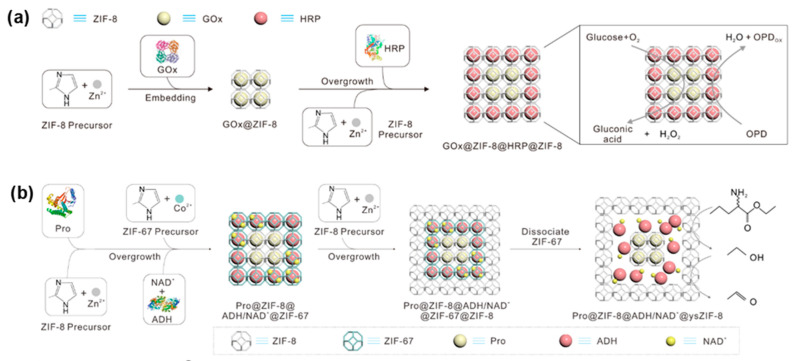
(**a**) Positional multi-step enzymes co-immobilization involving Gox and HRP in ZIF-8 and (**b**) positional multi-step enzymes co-immobilization involving alkaline protease and alcohol dehydrogenase in ZIF-67 and ZIF-8 (reprinted with permission from [86], Copyright 2022, Springer Nature).

A multi-step enzymes immobilization using zeolite imidazole frameworks (ZIF-8) was reported by Man et al. In detail, GOx was encapsulated in a ZIF-8 structure using the co-precipitation method. Then, the as-synthesized GOx@ZIF-8 performed two synthetic routes with HRP and other ZIF-8 precursors via a similar method. In this case, the GOx@ZIF-8 acted as a core for the growth of HRP@ZIF-8 which acted as a shell, in a core–shell structure, namely, GOx@ZIF-8 in HRP@ZIF-8. Despite that the synthesizing process is layer-over-layer assembly, the pore distribution of the original ZIF-8 did not change significantly; thus, the diffusion of the substrate through the ZIF-8 micropores was not restricted. As can be seen in Figure 8a, in the cascade reaction mechanism, the glucose molecule diffuses via the micropore of ZIF-8 and reaches the encapsulated GOx inside it, and then the produced H_2_O_2_ from this reaction is used by the encapsulated HRP in the outer side (shell) to catalyze the reaction of *o*-phenylenediamine (OPD) to 2,3-diaminophenazine (DAP), which can be detected fluorescently [86]. The main advantage of utilizing this enzymes immobilization strategy is to separate enzymes that cannot directly interact with each other but can carry out a cascade reaction. In the same work, they immobilize alkaline protease (Pro), ADH, and a co-enzyme (NADH) in different compartments. The ZIF-8 structure is shown in Figure 8b. This strategy is beneficial for preventing ADH digestion by the protease as well as maintaining NAD^+^/NADH co-factor regeneration.

Liu et al. presented a compartmentally immobilized enzyme system for the cascade reaction of glucose and phenol sensing. In this work, they immobilized HRP in the inner cavity of a hollow ZIF-8 structure, while the GOx was immobilized on the outer side, closer to the surface. A hollow ZIF-8 structure was achieved via a templated synthesis strategy using sodium deoxycholate (NADC) hydrogel. This strategy successfully overcomes the toxic effect of H_2_O_2_ for the enzyme. The sensing mechanism is based on the color change of ABTS in the presence of H_2_O_2_ from the glucose–GOx reaction and HRP. For phenol detection, the phenol substitutes for the role of ABTS in the reaction system. Another approach in multi-step enzymes co-immobilization is to separately immobilize enzymes in different MOFs and then mix them to carry out the desired cascade reaction. As an example, Xu et al. immobilized GOx and protease in different MOF carriers via separate immobilization processes. This strategy successfully prevents the digestion of GOx by protease and carries out a cascade reaction initiated by β-glucopyranose at the same time. In the synthesis process, a PMMA (polymethyl methacrylate) polymer was used to encapsulate the enzyme and forming a PMMA–enzyme spheres, and then UIO-66, a member of the MOF family composed from Zr^4+^ metal ion and benzene dicarboxylic acid (BDC) linker, was grown surrounding the PMMA–enzyme spheres. Zhao et al. reported a multi-step co-immobilization of cholesterol oxidase (ChOx) via surface adsorption and HRP via the pore entrapment method, on the same PCN-333 MOF. In the synthesis process, the PCN-333 was first synthesized using triazine-2,4,6-triyl-tribenzoic acid (H_3_TATB) ligand and Al^3+^ as metal ions. A short comparison of enzymes immobilization for cascade reactions in MOFs using one-step and multi-step immobilization strategies is shown in Table 3.

The ChOx was absorbed on the surface of PCN-333 via 40-min incubation at 37 °C. The HRP was then immobilized using a similar step on the ChOx/PCN-333. However, due to the dimension of HRP (4.0 nm × 4.4 nm × 6.8 nm) being smaller than that of ChOx (size: 5.13 nm × 6.30 nm × 7.30 nm), the HRP can penetrate and then be entrapped in the PCN-333 pores (pore size = 5.5 mm). This strategy can carry out the colorimetric detection of cholesterol in the presence of ABTS using a colorimetric approach [87].

### 4.3. Progress in MOF Cascade Enzyme in Electrochemical BFCs and Biosensor Applications

**Figure 9:**
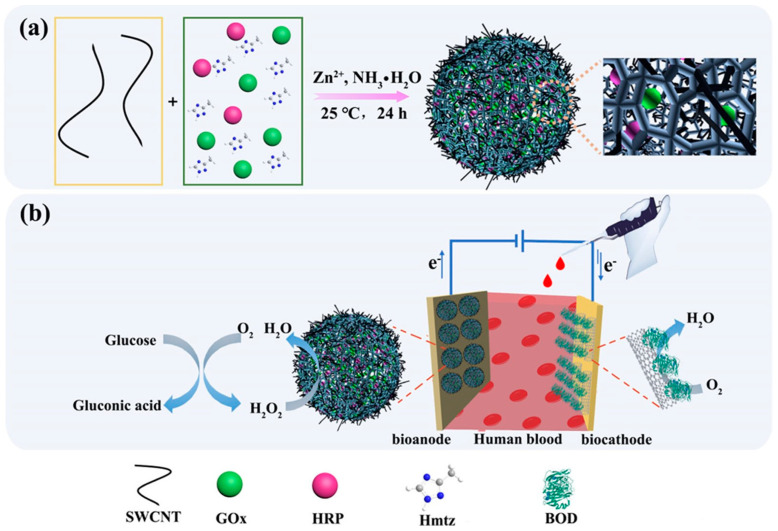
(**a**) synthesis process of SWCNT-MAF-7-GOx/HRP and (**b**)The construction of a BFC system involving SWCNT-MAF-7-GOx/HRP (reprinted with permission from [88], Copyright 2020, American Chemical Society).

Most of the reported works utilize MOFs to support cascade reactions of multi-enzyme in colorimetric or luminescence-based analysis. Only limited studies use this approach for electrochemical applications such as electrochemical sensors or BFCs. Compared to the colorimetric method, bioanalyte detection using an electrochemical technique provides some advantages such as wider linear range of detection and faster response time. Electrochemical techniques also can be combined with future technology such as wearable devices, self-powered devices, etc. For instance, Yimamumaimaiti et al. synthesized GOx–HRP encapsulated in MAF-7 (zinc and 3-methyl-1,2,4-triazole (Hmtz)) combined single-walled carbon nanotube (SWCNT) composite as a bioanode material for implantable enzymatic glucose BFCs. The encapsulation in an MOF could protect the enzymes against various inhibitors that present in human blood. The synergetic reaction of the GOx–HRP produces a high current density of 1.68 mA cm^−2^ at −0.35 V [88], as seen in Figure 9a,b. Meanwhile, when only GOx was used, the current density output was 0.3 mA cm^−2^. As shown in Table 4, the use of MAF-7 improves the enzyme bioelectrode stability when it is exposed with high temperature and some enzyme denaturant chemicals. This strategy increases the power density of the enzymatic BFCs in human whole blood from 14 µW cm^−2^ to 119 µW cm^−2^ compared to the unencapsulated enzymes.

More recently, an enzyme microneedle patch utilized encapsulated enzymes (GOx and HRP) in a ZIF-8 structure to construct an implantable self-powered device for diabetic wounds healing. A conductive polymer, polypyrrole (PPy), was used to improve the microneedle patch conductivity. The cascade reaction of GOx and HRP consumes the glucose in the wounds area of diabetes patients and produces a stable microcurrent that could improve the regeneration of the tissue around the wound area without leaving a tissue scar. The utilization of ZIF-8 in this work is also to protect the enzyme and to improve enzyme stability in human body fluid environment. In addition, ZIF-8 also has an antibacterial feature that can protect the wounds from microorganism infections [89].

Another group demonstrated that GOx was encapsulated with nickel palladium catalyst nanoparticles (NiPdNps) in a ZIF-8 nanoflower to obtain a nanobio composite material for glucose sensing applications. As reported, this strategy was able to detect glucose via both colorimetry and the electrochemical method. The NiPd acts as a tandem catalyst for the GOx. This material provides peroxidase-like activity and ORR activity, as well. In the colorimetry analysis, the peroxidase-like activity of the NiPd could oxidize the OPD in the presence of H_2_O_2_ from a glucose oxidation reaction, resulting in the color changing linearly with the increase in glucose concentration, while in electrochemical analysis, the ORR activity of the NiPd catalyst is more dominant. Thus, upon the glucose oxidation reaction by the GOx, the oxygen content in the solution will decrease, resulting in the decrease in the ORR current linearly with the increase in glucose concentration. These two methods have a significant difference in the saturation value of detection. By using the electrochemical technique, the dynamic range concentration obtained for glucose was 0.1 to 1.7 mM, while for the same case when the colorimetric technique was used, the dynamic range was observed to be 0.01 to 0.3 mM. Hence, the superiority of the electrochemical method over the other techniques is clear in this comparison [90].

## 5. Crosslinked Support

**Scheme 2:**
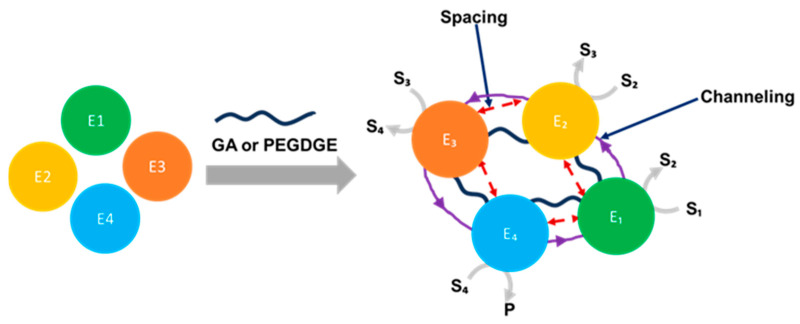
Immobilization of multi-enzyme via crosslinker and effects of spacing and channeling.

**Scheme 3:**
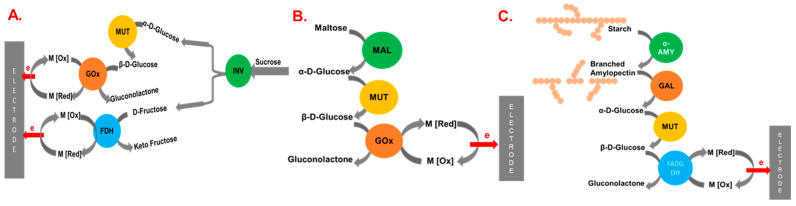
Immobilization of multi-enzyme via crosslinker and electron transfer mechanism in the cascade system for consumption of (**A**) sucrose [36], (**B**) maltose [38], and (**C**) starch [102].

It is easy to oxidize monosaccharides, such as glucose, to make a BFC using one enzyme [91,92]; however, disaccharides, such as sucrose, are challenging to oxidize using multiple enzymes, considering their highly efficient energy storage and biosensors [93]. A variety of research methodologies have been explored for the design of enzyme cascades, including programmed DNA scaffolds and random co-immobilization. It is usually easier to design an enzyme cascade system through random immobilization, such as adsorption, covalent binding, and crosslinking. Among these, crosslinking is the most promising technique that allows for the incorporation of enzymes and redox molecules onto the support platform [94]. In order to create the enzyme cascade system, the crosslinking technique is employed to co-immobilize different enzymes on the support surface with close proximity, thus allowing for its catalytic efficiency and reusability [95]. In addition, in some cases, the crosslinking technique can also control spatial distance and substrate channeling to attain catalytic efficiency [96]. Substrate channeling has a great impact on the design of multi-enzyme cascades to create an effective substrate oxidation pathway. This can be achieved via a co-immobilized enzyme, where the substrate transfers from one enzyme to another enzyme via the control of crosslinker spacer arm length (Scheme 2). The development of an optimal protocol considering the length and reactivity of a crosslinker for making an efficient cascade system for the implementation of biosensors and BFCs is therefore imperative. There are some examples of the use of crosslinkers for the design of cascade systems, for example, the Komaba group extensively investigated via a crosslinker in order to design a multi-enzyme cascade system, where sucrose, maltose, and starch were used as fuel for constructing BFCs. Handa et al. utilized glutaraldehyde (GA) for the co-immobilization of four different enzymes such as INV, mutarotase (MUT), GOx, and fructose dehydrogenase (FDH) in order to generate a sucrose BFC [36,97], as in Scheme 3A. The power density was generated by constructing the sucrose/O_2_ BFC as 2.9 mW cm^−2^. This work demonstrated a four-enzyme cascade system using sucrose as a fuel from beverages when the enzyme was co-immobilized via a crosslinker. The GA is widely used as a crosslinker for protein or enzyme conjugation [98], and the length of the crosslinker is very short (7.3 Å) [99]. However, the reaction of this crosslinker is prompt and makes a rigid multi-enzyme network [98]; such type of structure impedes the diffusion of a substrate through the multi-enzyme cascade network [100,101]. The same crosslinker GA, utilized by Yasujima et al., demonstrated BFCs to consume maltose as fuel for power generation by incorporating MAL, MUT, and GOx enzymes [38], as in Scheme 3B. This work successfully combines various enzymes to construct a BFC on carbon felt towards the developed BFC, demonstrating a high power output density of 2.3 mW cm^−2^ by design of maltose/O_2_ BFC. Considering the drawbacks of GA, alternatively, Toda et al. used poly(ethylene glycol) diglycidyl ether (PEGDGE) as a crosslinker to develop a bioanode using the co-immobilization of AA, glucoamylase (GAL), MUT, and flavine adenine dinucleotide-dependent glucose dehydrogenase (FAD-GDH), as in Scheme 3C [102]. This work successfully oxidized polysaccharide starch to the generation of a current via the co-immobilized multiple enzymes cascade system at 5.8 mA cm^−2^. The PEGDGE is a well-known protein/enzyme crosslinker, and the rate of reaction with the primary amine is slower; however, the spacer length is as high as 40 Å, which has a great impact on enzyme-to-enzyme spacing for the design of a multi-enzyme cascade system. The Minteer group developed a BFC where the mitochondrial enzyme was immobilized via two types of crosslinkers, GA and dimethyl suberimidate (DMS) [103]. The result exhibited a higher power density output (0.03 mW cm^−2^) via the crosslinked enzymes than via the free enzymes from the lysate of native mitochondria. Their finding mainly related to the positive effect on the substrate channeling that was performed by the crosslinker. The comparison of various BFCs is constructed using an enzyme cascade system via a crosslinker in Table 5. Considering the spacing among the enzymes on the cascade system, the length and reactivity of the crosslinker are important factors; therefore, an optimal-size and reactive crosslinker is desirable for the design of a cascade for biofuel and biosensors. In addition, the above-mentioned multi-enzyme cascade system immobilized by a crosslinker uses either GOx or FAD-GDH in order to oxidize the final substrate β-D-glucose to gluconolactone (Scheme 3) for the BFC or biosensor. Typically, in mediated electron transfer, the GOx can also utilize oxygen as an artificial electron acceptor [104]; therefore, the GOx cascade system may lead to the error of the biofuel and biosensor devices. Alternatively, FAD-GDH is the oxygen-insensitive prominent oxidoreductase enzyme employed for glucose oxidation. However, the FAD-GDH active center is covered by insulating debris that is unable to transfer electrons directly into the electrode surface; therefore, a redox mediator is required. In order to attain the stable BFC and sensitive biosensor, the FAD-GDH must be immobilized along with the redox mediator in the enzyme electrode system [105,106,107]. Table 5 provides the comparison of BFC based on a multi-enzyme cascade via crosslinkers of various modifications of anodes.

## 6. Polymeric Matrix/Hydrogel

**Scheme 4:**
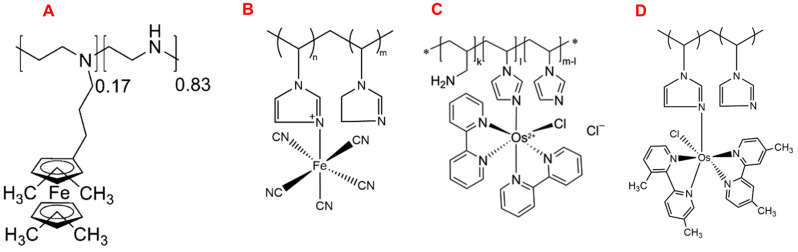
Redox polymer employed for enzyme cascade biofuel and biosensor: (**A**) Fc-LPEI [97,116], (**B**) cyanoferrate complex [118] (**C**,**D**) Os complex [108,121].

**Figure 10:**
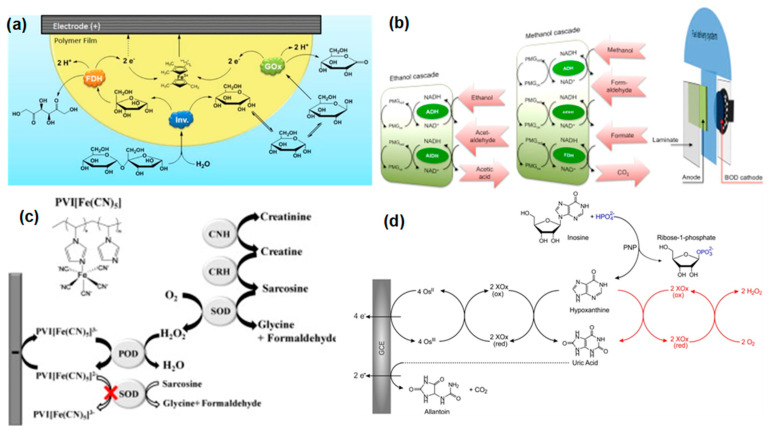
Electron transfer of multi-enzyme with redox polymer: (**a**) Fc-LPEI (reprinted with permission from [116], Copyright 2017, American Chemical Society), (**b**) LPEI-EGDGE (reprinted with permission from [117], Copyright 2015, Elsevier), (**c**) cyanoferrate complex (reprinted with permission from [118], Copyright 2013, Elsevier), (**d**) Os complex (reprinted with permission from [121], Copyright 2018, Elsevier).

The mediated multi-enzyme cascade system has an advantage in improving the energy density and sensitive detection for biofuels and biosensors, respectively [109,110]. The immobilization of the mediator with enzyme is crucial for the complete oxidation of fuel or biomolecules to obtain an efficient cascade system. The redox polymer contains an electrochemically active species along with an enzyme and a crosslinker in order to link with polymer and enzyme, thus allowing for a 3D structure [107,111]. Once it forms a network, it increases the immobilized amount of enzyme and redox species in the electrode surface, where electron transfer is improved by self-exchange among the immobilized enzyme mediator to the electrode surface [112]. The redox polymer has been widely applied in the design of BFCs [113] and biosensors [114] for several decades. In addition, the redox polymer still has many modern applications, such as the design of self-powered sensors, wearable sensors, and implantable sensors [115]. Several examples were also provided for how redox polymers can be used to create multi-enzyme cascade BFCs and biosensors. For example, Hickey et al. demonstrated a tetramethyl-ferrocene-modified poly(ethylenimine) (FcMe_4_-C_3_-LPEI) (Scheme 4A) as a redox polymer along with a crosslinked multi-enzyme cascade system including INV, FDH, and Gox, as in Figure 10a [116]. A crosslinker ethylene glycol diglycidyl ether (EGDGE) is used to interact from enzyme to enzyme, enzyme to polymer, and mediator to enzyme, thus allowing for a 3D network. They successfully oxidized sucrose via multiple enzymes, generating 0.36 mA cm^−2^ and 0.06 mW cm^−2^ of current and power density. The low power generation may be attributed to the slow conversion of the mutarotation of α-glucose and low cell voltage. Lau et al. demonstrated a linear polyethylene imine (LPEI) along with EGDGE as a crosslinker and poly MG as a redox mediator, as seen in Figure 10b [117]. This work describes two types of cascade systems, one for ethanol oxidation via ADH and ALDH and another one for methanol oxidation via ADH, ALDH, and formate dehydrogenase (FDH) multi-enzyme. This work generated 0.04 and 0.01 mW cm^−2^ power density by utilizing ethanol and methanol as fuel, respectively. Molecularly, this multi-enzyme cascade system was designed by the epoxy crosslinker as an enzyme–enzyme and enzyme–polymer 3D crosslinked network, where the enzyme is incorporated. However, the mediator is electropolymerized on the surface, and the electrical connection among the enzymes on the 3D network is challenging. Neih et al. designed a pentacyanoferrate complex-based redox polymer (Scheme 4B) for the mediated electron in order to detect creatinine from multiple enzymes such as creatinine amidohydrolase (CNH), creatine amidohydrolase (CRH), sarcosine oxidase (SOD), and peroxidase (POD), as shown in Figure 10c [118]. The redox complex successfully detected 12 µM of creatinine with 12–500 µM linear range. The 3D redox composite was prepared via a PEGDGE crosslinker, which incorporated enzyme along with redox polymer. Auino Neto et al. used a ferrocene-LPEI-based redox polymer with EGDGE as a crosslinker for the generation of power from ethanol via the immobilization of a multi-enzyme cascade system [97]. This work generated 0.02 mW cm^−2^ power density when ADH and ALDH were used and 0.01 mW cm^−2^ power density when ADH, ALDH, S-acetyl-CoA synthetase, citrate synthase, aconitase, and isocitric dehydrogenase were used. In this immobilization technique, the EGDGE is connected to enzyme–enzyme, enzyme–polymer, and polymer–polymer via the amine–epoxy reaction, which forms a 3D network with electron transfer by self-exchange via a mediator. Usually, the ferrocene has high formal potential and has some drawbacks, such as the oxidized form having slow hydrolysis and its derivatives not being soluble in aqueous solution, which causes an error in the design of sensors or BFCs [119]. Alternatively, the osmium (Os)-based redox complex easily controls the redox potential by changing ligands [120] and high solubility in water. Therefore, Kopiece et al. designed an Os complex with PEGDGE as a crosslinker for the immobilization of purine nucleoside phosphorylase (PNP) and xanthine oxidase (XOx) in order to detect phosphate molecules, as shown in Figure 10d [121]. The PNP and XOx were crosslinked in the redox polymer Os complex (Scheme 4C) via PEGDGE integration, where a 3D support matrix was formed via a PNP/XOx-to-Os complex and PNP-to-XOx through an amine–epoxy reaction. This design improves the bienzymatic layer, which allows the electron to shuttle from the enzyme to the electrode surface via a hopping mechanism. Recently, Rafighi et al. co-immobilized pyranose dehydrogenase (PDH) and *Rhodothermus marinus* β-glucosidase (*Rm*Bgl3B) on the Os complex-based buckypaper electrode in order to construct a β-glucan/O_2_ BFC. In molecular design, the GA was used to make a 3D network matrix with Os complex (Scheme 4D), wherein GA formed the PDH-to-*Rm*Bgl3B network along with the polymer matrix, which incorporated the enzyme and improved electron transfer. The β-glucan/O_2_ BFC generated 0.031 mW cm^−2^ power density. All the above-mentioned cascade systems are designed via a mediator tethered to the polymer backbone (Table 6). The electron diffusion for the mediator-tethered system can be described by a function of mediator amount, mediator flexibility, and self-exchange rate. In addition, the rate of electron transfer can be achieved by the extended spacer length from the mediator to the polymer backbone [122,123,124]. Therefore, an alternative immobilization technique is desirable in order to avoid the polymer-bound strategy such as a redox-crosslinked network into the multi-enzyme cascade system [94].

## 7. Conclusions and Future Perspectives

This review offers a comprehensive exploration of various materials, including nanosized materials, biomaterials, and micro/mesosized porous materials. The primary objective is to augment the efficiency of multi-enzyme cascade systems. Throughout this analysis, two critical factors consistently emerge: (1) the precise spatial arrangement of enzymes and (2) the effective channeling of substrates between these enzymes. These factors are pivotal in ensuring the seamless transfer of substrates between enzymes, minimizing undesired side reactions and ultimately amplifying the overall catalytic efficiency.

Selecting the most suitable nanomaterial for the intended application and consistently synthesizing it with the requisite properties pose formidable challenges that demand expertise in materials science. The task of affixing an enzyme cascade system to a nanomaterial while preserving enzyme activity and stability is particularly intricate. In intricate enzymatic systems, where factors beyond the target analyte can influence biosensor performance, acknowledging the crosslinking effect becomes imperative.

The integration of enzyme cascade systems with graphene oxide (GO) is an emerging research area, albeit one with a limited body of work compared to more established domains. It is evident that achieving a more profound or complete oxidation of fuels can significantly enhance the power density and current density of BFCs through the incorporation of enzyme cascade systems. However, the introduction of multiple enzymes into BFC construction introduces complexities that may potentially restrict its practicality. The utilization of multiple enzymes often enables the electrochemical detection of a greater variety of biomarkers and bolsters the performance of biosensors, but employing more than two enzymes in a solution can present challenges, such as random enzyme dispersion and collisions, introducing additional hurdles.

Thus, effective solutions necessitate interdisciplinary collaboration, cutting-edge nanomaterial fabrication techniques, thorough characterization protocols, and innovative biosensor design strategies. Future research endeavors will be dedicated to elevating the sensitivity and selectivity of enzyme cascade techniques built upon nanomaterial foundations.

## Figures and Tables

**Table 1:** Porous carbon materials and CNT-based enzyme cascade BFCs and biosensors.

Cascade System	Substrate	Immobilization Matrix	Power (μW/cm^2^)	Concentration (mM)	Ref.
LOx/POx	lactate	MgOC	1750	200	[25]
GA/GOD	starch	MWCNTs	8.15	0.5% (*w*/*w*)	[31]
INV/MUT/GOD/FAD	sucrose	MWCNTs-modified carbon felt (CF)	2900	500	[36]
INV/GDH	sucrose	MWCNTs	405 ± 6	200	[32]
ADH/ALDH/ForDH	methanol	carbon nanodots	68.7 ± 0.4	100	[37]
MAL/MUT/GOx	maltose	MWCNT-modified CF	2300	50	[38]

**Table 2:** Comparison of BFCs and biosensors based on multi-enzyme cascade system via DNA scaffold.

Cascade System	Fuel or Substrate	DNA Scaffold Structure	Power (μW cm^−2^)	LOD/Detection Range	Fuel Concentration (mM)	Ref.
INV/GOx	sucrose	DNA template	22	−	50	[78]
ALD/DLD	methanol	DNA template	24	−	120	[75]
GOx/HRP	glucose	RCA DNA flower	−	3 µM		[77]
GOx/HRP	thrombin	tetrahedron (TDN)-lattice	−	0.32 pM/0.001–10 nM		[70]
GOx/HRP	DNA	Tetrahedron (TDN)		3 fM/0.01 pM–10 nM		[79]

**Table 3:** Comparison of one-step and multi-step enzyme co-immobilization in MOFs.

Immobilization Techniques	Description	Advantages	Disadvantages
One-step	The enzymes were immobilized at once.	Simple synthesis process Short distance of each enzyme will improve the cascade reaction efficiency	Cannot be used to immobilize unmatched enzyme
Multi-step	The first enzyme was immobilized, followed by the immobilization of the second enzyme, the third enzyme, and soon in a compartment structure.	Can immobilize various types of enzymes, even unmatched enzymes or enzyme that can inhibit each other’s activity Prevent the toxicity of the enzyme caused by another enzyme’s byproduct reaction	Longer synthesis steps Limiting analyte diffusion

**Table 4:** Comparison of BFC performance with and without MAF-7 [88].

Aspects	SWCNT-GOx/HRP	SWCNT-MAF-7-GOx/HRP
Current density (mA cm^−2^)	0.3	1.68
Temperature stability (remaining current density after heating at 65 °C)	13%	53%
Chemical stability (remaining current density in organic solvents)	Significant current density loss when exposed to organic solvents	77% in THF 60% in methanol 60% in DMF 53% in pyridine
*P*_max_ in blood (μW cm^−2^)	14	119
OCV in blood (V)	0.22	0.34
Operation stability (hours)	2	14

**Table 5:** Comparison of BFC based on multi-enzyme cascade via crosslinker.

Multi-Enzyme as Anode	Fuel	Crosslinker	Current Output (mA cm^−2^)	Power (mW cm^−2^)	OCV (V)	Fuel Conc.	Ref.
INV, MUT, GOx, and FDH	Sucrose	GA	12	2.9	0.69	50 mM	[36]
MAL, MUT, and GOx	Maltose	GA	6.5	2.3	0.69	50 mM	[38]
AA, GluA, MUT, and FAD-GDH	Starch	PEGDGE	5.8	−	−	0.8% (*w*/*v*)	[102]
β-glucosidase and PDH	β-glucan	GA	−	0.031	−	0.5 % (*w*/*v*)	[108]
Metabolon	Pyruvate	GA, DMS	0.1	0.03	−	100 mM	[103]

**Table 6:** Enzyme cascade system based on redox polymer.

Multi-Enzyme as Anode	Fuel	Redox Polymer	Current Output (mA cm^−2^)	Power (mW cm^−2^)	OCV (V)	Fuel Conc.	Ref.
β-glucosidase, PDH	β-glucan	Os complex, glutaraldehyde	−	0.031	−	0.5 % (*w*/*v*)	[108]
INV, FDH, GOx	Sucrose	Fc-LPEI, EGDGE	0.36	0.06	−	100 mM	[116]
ADH, ALDH	Ethanol	Fc-LPEI, EGDGE	0.23	0.02	−	30 mM	[97]
ADH, AldDH, S-acetyl-CoA synthetase, citrate synthase, aconitase, and isocitric dehydrogenase	Ethanol	Fc-LPEI, EGDGE	0.12	0.01	−	30 mM	
ADH, AldDH	Ethanol	Poly-MG, LPEI-EGDGE	0.12	0.04	0.53	100 mM	[117]
ADH, AldDH, ForDH	Methanol		0.08	0.01	0.38	100 mM	
PNP, XOx	Phosphate	Os complex, PEGDGE	−	−	−	−	[121]

## Data Availability

The data will be made available on request.

## Abbreviations

AA	α-amylase.
ABTS	(2:2′-azino-bis(3-ethylbenzothiazoline-6-sulfonic acid)).
ADH	alcohol dehydrogenase.
ALDH	aldehyde dehydrogenase.
BDC	benzene dicarboxylic acid.
BFCs	biofuel devices.
BOD	bilirubin oxidase.
BSA	bovine serum albumin.
CC	carbon cloth.
CEL	cellulase.
CMC	carboxymethyl cellulose.
CNH	creatinine amidohydrolase.
CNT	carbon nanotubes.
CRGO	chemically reduced graphene oxide.
ChOx	cholesterol oxidase.
DAP	2:3-diaminophenazine.
DET	direct electron transfer.
DF	DNA flower.
DMS	dimethyl suberimidate.
DNA	deoxyribonucleic acid.
EGDGE	ethylene glycol diglycidyl ether.
FAD	flavin adenine dinucleotide.
FAD-GDH	flavin adenine dinucleotide-dependent glucose dehydrogenase.
FDH	fructose dehydrogenase.
ForDH	formate dehydrogenase.
GA	glutaraldehyde.
GCE	glassy carbon electrode.
GDH	glucose dehydrogenase.
GO	graphene oxide.
GOD/GOx	glucose oxidase.
GluA/GAL	glucoamylase.
H_3_TATB	2:4,6-triyl-tribenzoic acid.
HRP	horseradish peroxidase.
INV	invertase.
LOD	limit of detection.
LOx	lactate oxidase.
LDH	lactate dehydrogenase.
MAF-7	zinc and 3-methyl-1,2,4-triazole (Hmtz)).
MAL	maltase.
MG	methylene green.
MOF	metal–organic frameworks.
MUT	mutarotase.
MWCNT	multiwalled carbon nanotubes.
MgOC	magnesium oxide templated carbon.
NADC	sodium deoxycholate.
NADH	nicotinamide adenine dinucleotide.
NiPdNPs	nickel palladium nanoparticles.
NiR	nitrite reductase.
OCV	open circuit voltage.
OPD	*o*-phenylenediamine.
ORR	oxygen reduction reaction.
PADD	poly-(acrylamide-co-diallyl dimethylammonium chloride).
PC	porous carbon.
PDC	pyruvate decarboxylase.
PDH	pyranose dehydrogenase.
PEDGE	poly(ethylene glycol) diglycidyl ether.
PMMA	polymethyl methacrylate/poly(methyl 2-methylpropenoate).
PNP	purine nucleoside phosphorylase.
POD	peroxidase.
Pox	pyruvate oxidase.
PPy	polypyrrole.
RCA	rolling circle amplification.
RmBgl3B	Rhodothermus marinus β-glucosidase.
SOx/SOD	sarcosine oxidase.
SWCNT	single-walled carbon nanotubes.
TDN	tetrahedron.
TMB	3:3′:5,5′-tetramethylbenzidine.
XOx	xanthine oxidase.
ZIF	zeolitic imidazolate framework.
β-Gal	β-galactosidase.
